# H5N1 Clade 2.3.4.4b: Evolution, Global Spread, and Host Range Expansion

**DOI:** 10.3390/pathogens14090929

**Published:** 2025-09-15

**Authors:** Klaudia Chrzastek, Carolin M. Lieber, Richard K. Plemper

**Affiliations:** 1 Department of Population Health, College of Veterinary Medicine, University of Georgia, Athens, GA 30602, USA; 2 Center for Translational Antiviral Research, Institute for Biomedical Sciences, Georgia State University, Atlanta, GA 30303, USA; clieber1@gsu.edu (C.M.L.); rplemper@gsu.edu (R.K.P.)

**Keywords:** AIV, HPAIV, H5N1, clade 2.3.4.4b, mammalian infection, epidemiology

## Abstract

Highly pathogenic avian influenza viruses (HPAIVs) of the H5 subtype pose a continuous threat to animal and public health due to their zoonotic potential, rapid evolution, and ability to spread across continents. Since the emergence of the A/goose/Guangdong/1/96 (GsGD) H5 lineage in 1996, several clades have caused devastating outbreaks in poultry and wild bird populations, occasionally resulting in human infections. Of the many clades that have evolved, only three—clades 2.2, 2.3.2.1, and most recently 2.3.4.4b—have demonstrated the ability to spread globally. The 2.3.4.4b clade has raised significant concern due to its continuous geographic expansion, establishment in new ecosystems, including Antarctica, and increasing reports of mammalian infections, including companion animals, marine mammals, and livestock. Recently, cow-to-cow and cow-to-human transmission marked a paradigm shift in the epidemiology of avian influenza and emphasized the need for continued surveillance. This review summarizes the historical emergence, global spread, and molecular evolution of H5 HPAIVs with a specific focus on the recent expansion of clade 2.3.4.4b and its capacity for mammalian spillover.

## 1. Emergence of 2.3.4.4b Clade Highly Pathogenic Avian Influenza Viruses (HPAIVs)

Avian influenza viruses (AIVs) are classified as having either low pathogenicity (LP) or high pathogenicity (HP). LPAIVs generally cause mild infections, whilst HPAIVs can cause high rates of mortality in a wide range of avian species. AIV subtypes are defined based on their surface glycoproteins, hemagglutinin (HA; H1–H16) and neuraminidase (NA; N1–N9), and HPAIVs appear restricted to the H5 and H7 subtypes [1]. The influenza-like viruses of H17 and H18 subtypes were found in bats [2].

HPAIVs are of major concern for their pandemic potential and the socioeconomic impact of agricultural outbreaks. Specifically, the A/goose/Guangdong (GsGD) H5 HPAIVs, which emerged in 1996 in Southern China, are the only HPAIVs known to be sustained in wild waterfowl populations [3]. The H5N1 that infected humans in 1997 was confirmed to be a reassortant virus that acquired the HA gene from A/goose/Guangdong/1/96, the *NA* gene from A/teal/HongKong/W312/97 (Teal/HK/W312/97; H6N1)-like viruses, and the internal genes from A/quail/HongKong/G1/97 (Qa/HK/G1/97; H9N2-like) or Teal/HK/W312/97 viruses [4,5]. These viruses circulated for many years at a low level in aquatic poultry in China, until re-emerging with outbreaks in domestic chickens and associated human infections from 2003 onwards [6,7,8,9]. Driven by mutations and reassortment events, these viruses evolved rapidly. Genotypically, they were classified as A, B, D, E, V, W, X, Y, Z, and Z+ and spread globally [6,10]. In 2002, the Z-genotype was identified in China and spread next to Southeast Asia and towards the African continent in 2005. This was manifested by several reported outbreaks in Cambodia, Japan, Lao People’s Democratic Republic, Republic of Korea, Thailand, and Vietnam, with human infections detected in Thailand and Vietnam [10,11,12]. This virus was later defined as clade 2.2 and detected in Russia, Kazakhstan and parts of Europe, Egypt, India, and several western African countries where it has remained endemic in some countries (Bangladesh, Egypt, India, Indonesia, Vietnam and China, H5N1 subtype) [13,14,15,16,17,18,19,20]. This virus, once introduced to Egypt, diverged into a third-order clade, 2.2.1. The next substantial change in the Gs/GD epidemiology, was to establish two distinct lineages of virus, the 2.3.2 and 2.3.4 clades that co-circulated for some time in Asia and further diverged into distinct groups via genetic drift [13]. The 2.3.2.1 clade gained high prevalence in China and Southern Asia by 2008, and expanded into Mongolia, Russia, Eastern Europe, and to South Korea and Japan [13,21,22]. Contrary to the 2.3.2.1 clade, clade 2.3.4 did not expand outside Southern Asia (2008–2010) most likely due to its low association with wild birds [23,24]. After phylogenetic analysis of 2947 HA sequences of circulating strains, the WHO H5N1 Evolution Working Group consortium concluded that all circulating clades (clade 1 in the Mekong River Delta, 2.1.3 in Indonesia, 2.2 in India/Bangladesh, 2.2.1 in Egypt, 2.3.2, 2.3.4 and 7 in Asia) required assignment of divergent HA genes to new second-, third-, and/or fourth-order clades [13]. At the same time, clades 0, 3, 4, 5, 6, 8, 9, and several second- and third-order groups from clade 2 have not been detected since 2008 or earlier [13].

**Figure 1:**
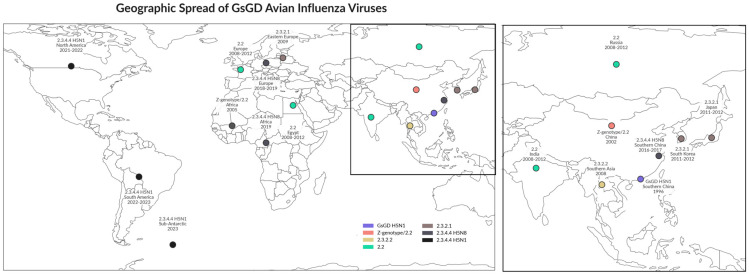
Geospatial distribution of A/goose/Guangdong (GsGD) of clade 2.2, clade 2.3.2.1, and clade 2.3.4.4 H5 HPAIVs. Those three clades of H5 AIV were able to spread globally since their first detection in 1996.

In the first decade of the 2000s, only two clades of GsGD viruses were able to spread across continents: clade 2.2 viruses from China to Europe and into Africa in 2005–2006 and clade 2.3.2.1 viruses from China to Eastern Europe in 2009–2010 (Figure 1).

In January 2014, domestic ducks were found HPAIV-positive near the Donglim reservoir in South Korea, and then also reported in breeder ducks and 100 dead Baikal teals [25]. The H5N8 HPAIV isolated from these samples, was a reassortant between the H5N8 virus detected in China in 2010 and other AIV circulating in China [25]. Detection of these viruses marked the start of a major outbreak of HPAIV H5N8, later categorized as clade 2.3.4.4, among poultry and wild birds in South Korea and China. Next, closely related HPAIV H5N8 were also reported in Japan in April 2014 [26]. First detection of HPAIV H5N8 2.3.4.4 in Europe was reported in Eurasian wigeon (Anas penelope) in Russia in September 2014 [27]. At the same time, these viruses also made their way to the United States and Canada in 2014, which was the first incursion of Gs/GD H5 viruses into the Americas.

In the US, H5N8 2.3.4.4 reassorted with locally circulating LPAIV, which resulted in the detection of a new HPAIV of the H5N2 subtype that contained five gene segments of the H5N8 virus and three from North American LPAIV and HPAIV H5N1 [28,29]. Afterwards, HPAIV H5N2 reassortant virus spread across poultry farms in the mid-western region of the United States in 2015. The widespread presence of HPAIV clade 2.3.4.4 in the US was confirmed by detection of these viruses in wild birds along the Pacific flyway from December 2014 until February 2015 [30]. In mid-2015, H5N8 clade 2.3.4.4 disappeared from Europe and North America; however, it was still circulating in Asia. A new reassortant of clade 2.3.4.4 H5N6 evolved and caused human infections [31,32,33]. Clade 2.3.4.4 H5N6 became endemic in China and Southeast Asia in 2013, reassorted with local AIV subtypes, and slowly replaced H5N1 viruses [34,35,36,37,38,39,40].

In 2016, HPAIV clade 2.3.4.4 reached Europe again [27]. HPAIV H5N8 was detected in a wild bird in the Uvs-Nuur Lake, Russia, in late May 2016 [27] and spread across Europe, the Middle East, and Africa, causing a large epidemic in 2016–2017. Until the end of 2019, sporadic detections and outbreaks were observed. These viruses are further divided into eight groups 2.3.4.4 a–h [41]. Clades 2.3.4.4a and d–h have circulated mainly in Asia, whilst 2.3.4.4b and 2.3.4.4c spread globally via migratory birds.

In 2019, H5N8 2.3.4.4b reached Africa, where it was found in Nigeria, Namibia, South Africa, and Egypt [42]. In December 2019, Poland reported the first detection of clade 2.3.4.4b H5N8 virus in 14-week-old meat turkeys, suggesting that the virus had reached Europe. Swieton et al. [43] have shown that the virus was generated by reassortment between the H5N8 from sub-Saharan Africa and LPAI viruses from Eurasia. This caused a total of 20 outbreaks in poultry in Poland by 31 January 2020. Similar outbreaks were then found in Slovakia, Hungary, Romania, Germany, and the Czech Republic [42].

## 2. Global Spread of H5N1 HPAIV Clade 2.3.4.4b Since 2020

The GsGD H5 lineages have consistently geographically expanded since 2020. Reassortment events between H5N8 and circulating local viruses led to a shift in the dominant virus subtype from H5N8 to H5N1 in 2021 [44]. By the end of 2021, 30 countries or territories across Asia, Europe, and Africa reported the detection of viruses of this clade in birds [41], and most of these outbreaks had been caused by H5N1 2.3.4.4b viruses. In total, since January 2022 to 29 December 2023, 2.3.4.4b H5N1 HPAI has been responsible for over 11,400 bird outbreaks across Africa, Asia, Europe, and North America [45].

### 2.1. Europe

In Europe, after confirmation of the 2.3.4.4b clade in Poland in December 2019, H5N1 was detected in 37 European countries in 2021–2022 (with a total number of outbreaks of 6684, of which 2761 were reported in domestic birds and 3923 in wild birds) [46]. This ongoing outbreak reached 4698 virus detections reported by 33 European countries on 23 June 2023 (1274 and 3424 cases in domestic and wild birds, respectively) between 3 December 2022 and 23 June 2023 [47]. Compared to the reporting period of December 2022–March 2023 (522 and 1138 outbreaks in domestic and wild birds, respectively), the prevalence slowly decreased over the spring and summer in domestic and wild birds in Europe as of September 2023 [46,47,48]. Overall, the cumulative number of outbreaks reported in September 2023 was 5419, which includes 1303 outbreaks in domestic birds and 4116 in the wild bird population. Based on the EFSA report (December 2023), which also started the new 2023–2024 epidemiological season, there were 263 outbreaks reported (88 in domestic birds and 175 outbreaks in wild birds) [47]. Between 7 December 2024 and 7 March 2025, 743 outbreaks were detected in poultry and wild birds, mostly waterfowl, such as mute swans, barnacle geese, and graylag geese [47]. The H5N5 outbreaks occurred only in wild birds in waterfowl.

In wild birds, high mortalities were observed in gull species, particularly in black-headed gulls (in France, Belgium, The Netherlands, and Italy) between December 2022 and March 2023, and continued through to September 2023, along with a new seabird species being infected (sandwich terns) [48]. Interestingly, common cranes (Grus grus) were the most frequently affected other wild bird species during September to December 2023 (*N* = 48), with high mortality rates being reported from Hungary and several other countries in Europe [48]. Previously, the virus was only found in feces of common cranes in China (2.3.4.4b H5N8) and birds in Israel (2.3.4.4b H5N1), where the virus killed almost 10,000 birds in Agamon Hula, which is part of the Hula Valley, a large water lake on the route of the Jordan River in North-East Israel [49,50]. Yang et al. [50] showed that H5N8 viruses in the feces from common cranes in China carried some mutations that contributed to a binding preference for the α-2, 6-linked human sialic acid galactose receptor in HA and mutations that increase polymerase activity in mammalian cells. However, no mutation in position 627 in the PB2 gene was found. The E627K substitution is a well-known adaptive mutations that enhance replication of AIV in mammalian cells [51,52]. The predominant genotype circulating in Europe from February to August 2023 was genotype BB, which accounted for almost 80% of samples tested in Europe during this period [48]. The BB genotype was found at high frequency among black-headed gulls and other gull species. Thereafter, the frequency of BB sharply declined (from September 2023), most probably due to different birds being infected at that time (Anseriformes versus seabirds and other new species, such as common cranes) [48]. Additionally, a new variant of the 2.3.4.4b virus was detected that acquired a PB1 segment from circulating LPAIV in the area, changing the epidemiological situation in Europe [53]. The genotypes BB, AB, CH, and I (H5N5) emerged in Europe in 2021 and were found in Norway, Iceland, and the United Kingdom. Viruses belonging to the CH genotype were found in poultry, domestic cats, storks, and caracals in Poland, and carried the PB2-E627K mutation that had not been identified in other EU countries [54]. In addition, seven reassortants were circulating in Europe: DA genotype (all common cranes), DB (isolated from the wild and domestic bird population in northern Europe), DC (H5N1-A/Common_Buzzard/Netherlands/23023642-002/2023-like), DD (H5N1-A/Pheasant/England/113705/2023-like), DE (H5N1-A/Chicken/Scotland/114176/2023-like), DF (H5N1-A/Sparrowhawk/Scotland/131359/2023-like), and DG (H5N1-A/Chicken/Germany-NI/2023AI08838/2023-like) [47].

Since December 2024, the dominant genotype has changed, and the majority of viruses in Europe now belong to genotype EA-2024-DI. They are mostly found in Anseriformes and domestic birds. Phylogenetically, these viruses form two genetic groups, DI.1 and DI.2, of which DI.2 is the most frequent (>90% of the EA-2024-DI viruses) and widespread across the European countries [55].

In the UK, circulating genotypes of H5N1 2.3.4.4b were in 2021–2022 mostly reassortants of Eurasian H5N1 with Eurasian AIVs (different H5Nx subtypes) [56]. Two sub-lineages of the AIV07 genotype, AIV07-B1 and AIV07-B2 460, were detected between October 2021 and May 2022, whereas the AIV08 genotype was only detected in a single poultry case (2021/2022). Further, three other genotypes were detected: AIV09, which showed high sequence identity with the AIV07 genotypes but contained different PB2 and PA genes (reassortment events in summer 2021); AIV20 (like AIV07-B2 genotype, except for their *NP* gene); and AIV55 [56]. As of 9 June 2025, there have been 63 confirmed cases of H5N1 HPAIV and one of H5N2 in the UK in poultry and captive birds in the current outbreak [57].

### 2.2. North and South America

In December 2021, viruses from the 2.3.4.4b clade reached Canada and were isolated from poultry and black-backed gull (Larus marinus) [58]. They were genetically related to viruses circulating in Europe in early 2021. These viruses were isolated from wild waterfowl from two Atlantic coastal states corresponding with the Atlantic flyway [59]. Next, HPAI (H5N1) has also infiltrated poultry farms throughout Canada, with 11,030,500 having been affected as of 6 May 2024 [60]. Viruses of the 2.3.4.4b clade were first detected in the US in January 2022. By 1 July 2024, over 97 million birds had been infected in 48 States [61].

In November 2022, HPAIV caused high mortality in Peruvian pelicans [48]. Sporadic H5N1 cases followed in pelicans from Panama, Honduras, Costa Rica, and Guatemala between December 2022 and February 2023. The virus was also detected in captive birds in Cuba, blue-and-white swallows in Bolivia, and swans in Uruguay. The first H5 cases in Argentina and Uruguay were reported in February and March 2023, respectively, after which the virus spread further across South America [62]. Chile and Colombia reported the first cases of A/H5N1 in March 2023, and Brazil in May 2023. Between September and December 2023, H5N1 was reported in Brazil (951 outbreaks, mostly in wild birds), Colombia (4 outbreaks), Costa Rica (1 outbreak), Ecuador (5 outbreaks), Peru (8 outbreaks), Uruguay (2 outbreaks), and Venezuela (1 outbreak). In June 2023, swab samples from the central nervous system of Royal terns and Cabot’s terns were collected in Brazil. From these samples, 4 H5N1 HPAIVs were detected via RT-qPCR, and complete genome sequences for TM/BR08/23 and TM/BR09/23 and partial sequences for TA/BR25/23 and TM/BR26/23 were obtained [63,64]. All H5N1 isolates possessed polybasic amino acid sequences at the HA cleavage site (PLREKRKKR/GLF) and shared high sequence identities (99.59–100%) across all the genes. Furthermore, they were also highly similar to H5N1 isolated from Chile and other South American countries, belonging to the B3.2 genotype, and carried reassortment type identified in the US in 2022. This B3.2 genotype contains PB2, PB1, NP, and NS originating from North American strains and PA, HA, NA, and M of a Eurasian origin [41,64]). Furthermore, no evidence of reassortment was observed, indicating that the viruses were direct descendants of B3.2.

### 2.3. Sub-Antarctic and Antarctic Region

The first case of 2.3.4.4b in the sub-Antarctic region (British overseas territory of South Georgia at Bird Island) was found in brown skuas on 8 October 2023 [65]. Later, Banyard et al. [65] confirmed clade 2.3.4.4b H5N1 HPAIV across four different sampling locations in South Georgia and in the Falkland Islands. The main affected bird species were brown skuas, where the mortality rate increased rapidly throughout one month. Furthermore, clinical disease was also manifested in elephant seals (Mirounga leonine) in South Georgia [65]. The viruses found in Bird Island and the Falkland Islands were classified as the B3.2 genotype that arose in early 2022 in North Dakota (US), which was then reported in South America between October 2022 and March 2023 [58,65,66]. Prior to October 2023, a large number of samples from a range of species and locations were collected from early November 2022 to late March 2023, comprising the entire austral breeding season in 2022–2023, and no HPAIV was found in the Antarctic region [67]. However, influenza A virus (H3N8, H1N1) was confirmed for the first time in coastal areas of Antarctica in samples collected from birds and marine mammals in April–May 2023 [68].

In March 2025, highly pathogenic H5N1 avian influenza virus was reported in 13 bird and mammal species across 24 of 27 surveyed sites in Antarctica [69]. Whole genome sequencing of samples collected from kelp gull (8 January 2024 at Hannah Point), pintado petrel (3 December 2024 at Hannah Point) and Antarctic fur seal (4 December 2024 at Robert Point, Robert Island) revealed that the viruses belong to the GsGD clade 2.3.4.4b with the B3.2 genotype; however, each of these three hosts descended from a different introduction event [70]. The gull and fur seal strains clustered with HPAIV H5N1 recovered from wildlife in the Atlantic coast of South America in October 2023 whereas the sequences of the viruses recovered from the pintado petrel on December 2024 clustered with those of an HPAIV recovered from a brown skua two weeks later at the Torgersen Island (~290 km southwest) and both were descendants of the HPAIV strains that circulated in South Georgia around October to December 2023 [70]. This suggests that there is extensive epidemiological connectivity between South America and Antarctica, with South Georgia being a region where many species come in contact and thus might serve as a mixing vessel for the virus spread in the region [70]. This also raises concern for the endemic wildlife whose mortality may go largely unrecorded due to limited surveillance in Antarctica, and indicates a risk of further viral incursions into Antarctica and potentially to currently unaffected regions such as Australia.

### 2.4. Australia

Australia has long been free from H5N1 HPAIV infection, facing multiple outbreaks of H7Nx in poultry (specifically involving H7N3, H7N9, and H7N8 strains) [71]. Although no HPAIV H5N1 was detected in animals in Australia, the first human case of H5N1 was confirmed on 18 May 2024, and the WHO was notified on 22 May. The H5N1 clade 2.3.2.1a was confirmed in a child who traveled back to Australia from India [72]. Genetic analysis has shown that it is a reassortant virus consisting of clade 2.3.2.1a, 2.3.4.4b, and wild bird low pathogenicity avian influenza gene segments [72].

## 3. H5N1 HPAIV Spillover to Mammals

Along with the outbreaks in avian species, spillover events of HPAIV H5N1 virus to mammals have been continuously reported. These cross-species transmissions have affected over 43 mammalian species spanning Europe, North America, South America, and Asia. Based on the WOAH report (as of May 2025), almost 1500 cases of H5N1 in different mammalian species were reported, some of them being fatal (mostly in marine mammals and fur animals). Interestingly, 1252 cases in mammalian species were reported by the US only, including positive samples from dairy cattle [73].

### 3.1. Avian Influenza in Cattle and Small Ruminants

A multi-state outbreak in the US of HPAI (H5N1) in dairy cows was reported on 25 March 2024, and four human cases were confirmed to be infected following exposure to dairy cattle [74,75]. This was the first recorded instance of mammal-to-human transmission. To date, 41 human cases of H5 HPAI have been reported, all linked to exposure associated with commercial cattle operations as of 15 August 2025 [76]. Infected cows showed detectable infectious virus and RNA in milk, and data suggests cow-to-cow transmission most likely involving contaminated milk and milking equipment; however, the exact mechanism is still being investigated [77]. Animals displayed a variety of clinical signs such as decreased feed intake, altered fecal consistency, respiratory distress, and decreased milk production with abnormal milk [77]. Clinically affected cows shed the virus more frequently in milk (24/25) compared to subclinical cases (1/15). In contrast, viral detection in nasal swabs and urine was lower among clinical cases (6/25 nasal swabs and 2/15 urine samples) than in subclinical cows, where the virus was detected in 6/19 nasal swabs and 4/8 urine samples. Alkie et al. [78] showed that complete inactivation of clade 2.3.4.4b H5N1 virus in milk that was spiked with 6.3 log_10_ EID_50_ was achieved after incubation of milk at 63 °C for 30 min and was also observed in seven of eight experimental replicates when treated at 72 °C for 15 s.

Interestingly, distinct tropism of HPAIV H5N1 between the cattle and cat samples was shown, where the virus was present in the mammary tissue of cattle and the central nervous system of cats [77]. Genetically, all samples from dairy cattle until February 2025 were classified within a newly emerging B3.13 genotype, originating from Eurasian wild bird ancestry (PA, HA, NA, and M gene segments) and American bird lineages (PB2, PB1, NP, and NS). The PB2 and NP segments of B3.13 genotype derived from LPAIV of H3 and H11 subtypes, and it was first detected on 25 January 2024 in a Canadian goose in Wyoming, followed by detection in a peregrine falcon in California and a skunk in New Mexico in February 2024 [77]. A new H5N1 genotype, designated D1.1, was identified in cattle through the National Milk Testing Strategy (NMTS) surveillance program in the United States. This virus is a 4:4 reassortant that retained PB1, HA, MP, and NS segments from the Eurasian avian lineage and acquired PB2, PA, NP, and, most notably, N1 from LPAIV circulating in the Americas [79].

In March and May 2024, the H5N1 virus was also detected in goat and alpacas by the USDA’s National Veterinary Services Laboratories (NVSL), respectively. The newborn goat was exposed to the B3.6 genotype, which was commonly found in North American wild birds and sporadically detected in poultry flocks in 2023 and 2024, whereas alpacas were infected with B3.13, the same virus strain detected in U.S. dairy cattle [80]. Furthermore, the first case of H5N1 HPAIV was also confirmed in sheep in Yorkshire, England, in March 2025 [57].

### 3.2. Avian Influenza in Seals and Fur Animals

Different subtypes of AIV were isolated from seals in the last 40 years [81]. In January 2024, Argentina reported mass mortality among young Southern elephant seal (*Mirounga leonina*) pups, which represented almost 96% of all seal pups born across Argentina in 2023 [82]. Previously, a mass mortality event of more than 3000 sea lions (*Otaria flavescens*) was observed in January and February 2023 in Peru [83]. The viruses responsible belong to the HPAI A/H5N1 lineage 2.3.4.4b and are 4:4 reassortants, in which PA, HA, NA, and MP belong to the Eurasian lineage that initially entered North America from Eurasia, and the remaining PB2, PB1, NP, and NS segments came from an American lineage that was already circulating in North America [66]. As reported by Leguia et al. [66], these viruses did not acquire mutations linked to mammalian host adaptation and enhanced transmission (such as PB2 E627K or D701N), but at least eight novel polymorphic sites were found in their genome. Occurrence of the H5 virus in unusual hosts, like *Otaria flavescens,* was also reported in Chile on 10 February 2023 [84]. Genetic characterization of isolates obtained from birds and marine mammals revealed that all Chilean H5N1 viruses belong to lineage 2.3.4.4b and cluster monophyletically with viruses from Peru, indicating a single introduction from North America into Peru/Chile [85]. The D701N (in two sea lions, one human, and one shorebird) and Q591K (human and one sea lion case) mutations were identified in PB2 segments [85]. Interestingly, a minor population of viruses carrying the D701N mutation was present in 52.9–70.9% of sequence reads obtained from the samples tested, suggesting a mixed population of viruses within the sample [85].

Previously, HPAIV A/H5N1 infection in mammals was reported in seals in the US (New England). After sequencing of 71 avian- and 13 seal-derived virus genomes from New England, in contrast to what was reported by Leguia et al. [66], all but one virus represented non-reassortant Eurasia 2.3.4.4b viruses [86]. The authors concluded that the virus outbreak among New England harbor and gray seals was concurrent with a wave of avian infections in the region, and the evidence of mammal adaptation existed in a small subset of seals (PB2 E627K or D701N mutations) [86].

HPAIV (H5N1) was also found in infected farmed American mink (*Neovison vison*) in Spain in October 2022 [87]. These viruses belong to HPAI clade 2.3.4.4b and cluster with A/gull/France/22P015977/2022-like genotype (gull/FR) (showing high [99.8–100%] similarity) [87]. This genotype was identified previously in wild birds (The Netherlands, Belgium, and France), and then in chicken and fox in Belgium [88]. A gull/FR-like genotype is a reassortant virus that showed *PA*, *NP*, and *NS* gene segments originating from gull-adapted H13 subtype. Aguero et al. [87] have shown that mink viruses carried eight–nine amino acid differences from the closely related H5N1 in their internal genes, and interestingly, all mink viruses carried alanine (A) at position 271 of PB2 (T271A), which was previously shown to enhance polymerase activity in mice [89]. Other single-nucleotide polymorphisms (SNIPs) were also detected in the polymerase (PB1-388R and PB1-F2–30L; PA-56T), neuraminidase (NA-74S and NA-163L), and non-structural (NS2–13G) segments [87]. The H5 clade 2.3.4.4b virus was also found in two foxes in the Netherlands and was related to other HPAI H5N1 viruses detected in wild birds and poultry in Europe during 2020–2021. Six amino acid differences (A152T and T521I in PB2; M644V in PB1; A336T in NP; L22S in NA and D209N) in the viral genome were reported [90]. Recently, the H5N1 virus was found in fur farms in Finland, where Arctic foxes, raccoon dogs, American mink, and red foxes were kept [91]. This outbreak started in mid-July 2023 and was caused by the 2.3.4.4b genotype BB [92]. Genetic analyses of the samples collected from farms have shown that some adaptational mutations were found in the genome, such as E627K and T271A in PB2 [92]. Based on epidemiological studies, it was possible that transmission between fur animals might have occurred (mortality on affected farms has been 2–4 times the normal rate and, at the peak of the outbreak, a large farm recorded almost 400 deaths in one day, which is 10 times the normal; however, the exact mechanism of transmission within or between the farms remains unknown [92]. Previously, the mortality of wild carnivores (an otter, two red foxes, and a lynx) caused by HPAIV 2.3.4.4b was reported by Finland in fall 2021; this event was connected with mass mortalities of farmed and released pheasants in the same area [93]. The viruses isolated from mammals and pheasants in Southern Finland clustered together [93]. Interestingly, all four isolates from mammals carried mutations in the PB2 segment (PB2-E627K and PB2-D701N) known to facilitate replication in mammals [93]. Previously, a high number of seropositive wild carnivores were reported in the Netherlands between 2020 and 2022 [94]. Virological evidence of HPAI H5 virus infection was found in 0.8%, 1.4%, and 9.9% of animals tested in 2020, 2021, and 2022, respectively, with the highest positive proportion in foxes, polecats, and stone martens [94].

### 3.3. Avian Influenza in Companion Animals

The H5N1 2.3.4.4.b virus is also responsible for the infection of companion animals [54,95]. Mortality in domestic cats infected with H5N1 HPAIV was reported by Poland and South Korea in June and July 2023, respectively [54,96]. Furthermore, feline infections were also recorded in Thailand (2004, 14 cases), Germany (2006, 3 cases), Austria (2006, 1 case), Iraq (2007, 2 cases), Egypt (2007, 2 cases), Russia (H5N8, 2016, 2 cases), France (H5N8, 2020, 1 case), and Italy [97,98]. Since mid-March 2024, the confirmed cases of H5 viruses in cats were constantly growing within the US and are connected with consumption of unpasteurized milk and raw or undercooked meat from affected animals (e.g., poultry) [99,100]. In the US, cats are mostly infected with the genotype B3.13 strain, the same strain that has been circulating among dairy cattle.

In Poland, 25 out of 46 samples collected from cats were positive for HPAIV and belonged to the genotype CH (H5N1 A/Eurasian wigeon/Netherlands/3/2022-like), which was previously seen in Poland between December 2022 and January 2023 [54]. Interestingly, the viruses isolated from cats in Poland possessed mutations in the PB2 protein (526R and 627K) that were previously detected in isolates from white storks, which were found dead in early June (mutation 627K in PB2) [54].

Genetic characterization of 2.3.4.4b H5N1 virus isolated from cats in South Korea showed a high degree of all eight sequence identities, ranging from 99.59 to 100%, with the HPAI H5N1 virus clade 2.3.4.4b isolated from birds in Japan between November 2022 and April 2023, indicating that the viruses that infected cats originated from migratory birds traveling from Japan and South Korea during the previous winter [96,101,102,103]. This isolate was classified later as South Korean genotype III and contained several mammalian adaptive mutations, which were also found within the avian population, except for D701K, which was only found in cat isolates [96]. Previously, in April 2023, Italy reported that five domestic dogs and one cat seroconverted to 2.3.4.4b H5N1 without showing any clinical signs of disease [104]. These animals were living on a backyard farm where an outbreak of clade 2.3.4.4b was reported in poultry [104]. Interestingly, the viruses isolated from hens differed from all other HPAI H5N1 clade 2.3.4.4b viruses circulating in poultry and in birds by a mutation in the PB2 protein, T271A, which was not observed in H5Nx viruses of clade 2.3.4.4b collected from birds in Europe since 2020. However, it was found in some virus populations isolated from mammals, e.g., in the viral genome from an outbreak on a mink farm in Spain and Finland [87,92,104]. The scale and dissemination of the viruses between infected animals in 2023 suggested productive mammal-to-mammal infection, which was later confirmed. Recent reports also propose that the viruses acquire mammalian adaptations at genomic levels, especially in the PB2 segment, either after infecting mammalian species or, interestingly, beforehand at the avian level, as was already shown, for example, in backyard hens in Italy [104] or white storks in Poland [54]. Kim et al. [105] have shown that PB2-E627K minor population of viruses carrying 627K in their PB2 segment (below 5%) can rapidly evolve following a single infection in mice, transmit, and reach nearly 100% in direct-contact mice, suggesting that even a small proportion of mammalian-adaptive mutations can quickly become dominant as the virus serially transmits between mammals.

Although infections of dogs with H5N1 clade 2.3.4.4b viruses have been rarely reported, antibodies to H5 and N1 have been detected in hunting dogs with frequent exposure to wild birds and waterfowl retrieval in both the U.S. and Thailand [106,107]. Additionally, Canada reported a fatal canine infection in April 2023, shortly after the dog chewed on a dead wild goose [108].

## 4. Human Cases of H5N1 Infection: Overview and Recent Developments

### 4.1. Cumulative Global Cases and Fatality

A cumulative number of 976 confirmed human cases of avian influenza A (H5N1) were reported from 25 countries to the WHO from 1 January 2003 to 27 May 2025, out of which 470 were fatal [109].

### 4.2. Recent Human Cases (2022–2025)

Until April 2024, all human cases of H5N1 clade 2.3.4.4b reported to WHO were associated with contact with infected birds. Between January 2022 and December 2023, 19 human cases were reported across eight countries: Ecuador (1 case, clade 2.3.4.4b), Chile (1 case, 2.3.4.4b), Cambodia (6 cases, clade 2.3.2.1c), China (2 cases, clade 2.4.4b), UK (5 cases, 2.3.4.4b), Spain (2 cases, 2.3.4.4b), US (1 case, 2.3.4.4b), and Vietnam (1 case, clade not reported) [110].

Along with the geographical expansion of H5N1 viruses via migratory birds, the first reported cases of human infection by avian influenza A (H5) virus were reported in Latin America starting on 9 January 2023 [111]. The clinical disease developed fast, and the person infected was admitted to the intensive care unit with septic shock and was treated with oseltamivir and mechanical ventilation due to pneumonia [111]. Followed by the human infection in Ecuador, the next case in Latin America was reported by Chile on 29 March 2023 [112]. The person developed severe illness and was admitted to the hospital, where they started treatment with oseltamivir and antibiotics. Genetic characterization of the virus showed that it belonged to the 2.3.4.4b clade and carried two mutations in the PB2 segment (D701N and Q591K) compared to the PB2 genome obtained from circulating wild birds in this area, suggesting that the virus acquired these mutations during the course of illness.

In February 2023, Cambodia notified the WHO of a confirmed case of A (H5N1) virus in two people from the Prey Veng province; one person developed severe pneumonia during the course of infection and died, and the second person had a mild influenza-like illness [113,114]. Virus sequencing showed that the A (H5N1) viruses from the cases belonged to the A (H5) genetic clade 2.3.2.1c and were similar to the 2.3.2.1c clade viruses circulating in poultry in Southeast Asia since 2014. In the same month (February 2023), two people from China tested positive for A (H5N1), clade 2.3.4.4b; both had been exposed to backyard poultry [114]. The next human infections were reported by Cambodia on 8–9 October 2023. A young girl developed severe clinical disease and was admitted to the hospital on 5 October and passed away one day later [115]. There were reports of dead and sick chickens in the village prior to her illness onset. The other person infected also had contact with sick and dead chickens before his illness onset [115]. The viruses were classified as A (H5N1) clade 2.3.2.1c, and the presence of the PB2 627K marker was identified [45]. Previously, one human infection was reported by the Vietnamese authority in October 2022, and this person lived in a household where backyard poultry was raised (chickens and ducks); this person was hospitalized and treated at an intensive care unit [115].

Between the start of 2024 and early 2025, 71 human cases were reported: 70 in the United States and one in Canada in November 2024 [116]. Of these, 41 cases in the U.S. were linked to exposure to sick or infected dairy cattle [116]. From 1 January to 4 August 2025, an additional 26 human infections were reported worldwide [117]. Recent reports highlight that Cambodia has seen 14 infections in 2025, with eight deaths (seven in children) caused by clade 2.3.2.1e circulating subtype. India confirmed two fatal cases belonging to clade 2.3.2.1a, while Mexico reported a fatal pediatric case tied to clade 2.3.4.4b, the same one circulating in North America [118]. Vietnam documented an infection in a child with encephalitis linked to clade 2.3.2.1e [117,118]. Additional cases were identified in Bangladesh, China, the United Kingdom, and the United States. These global infections emphasize the critical need for robust influenza surveillance and preparedness, with candidate vaccines already under development to target the most relevant circulating clades.

### 4.3. Paradigm Shift: Mammal-to-Human Transmission

The first human infection associated with bovine-origin H5N1 in the United States was reported in Texas, following multiple outbreaks in dairy cattle that occurred in March 2024. This case marked the first recorded instance of mammal to human transmission [119]. Uyeki et al. [120] showed that the virus isolated from both the conjunctival and nasopharyngeal swab specimens belonged to clade 2.3.4.4b (genotype B3.13), and all gene segments were closely related to viruses detected in cattle. Genetic characterization demonstrated that viral sequences from cattle and farm workers maintained primarily avian characteristics and lacked changes in the HA gene segment. However, the human sample contained an E627K substitution in the PB2 segment. No genetic markers associated with reduced susceptibility to influenza antiviral drugs were found [120].

By August 2025, 41 human cases associated with dairy cows were confirmed in the United States. Human infections with bovine-origin H5N1 (B3.13) typically presented with mild symptoms, including conjunctivitis and coughing [120]. The second H5N1 genotype detected in United States cattle was a clade 2.3.4.4b, genotype D1.1 virus that was discovered through the NMTS testing program, and confirmed on 31 January 2025 [100]. Interestingly, a fatal case involving a person infected with H5N1 occurred in the United States. To date, the United States has reported one fatal case on 6 January 2025 that involved a person infected with H5N1. This infection was attributed to H5N1 2.3.4.4b genotype D1.1, which had previously been linked to outbreaks in wild birds and poultry [121]. A severe case involving a D1.1-infected patient was also reported by Canada, indicating high pathogenicity of this genotype for humans [122]. Genetic characterization of B3.13 and D1.1 genotypes revealed that these two groups of viruses may have differing reassortant origins and host adaptation profiles [123]. The B3.13 has acquired mammalian adaptation mutations (e.g., HA–137A, HA–160A) that might enhance human receptor binding capabilities of the viruses, whereas D1.1 remains predominantly avian-adapted and linked to poultry as a transmission source with limited mammalian input. An exception is cats, which were infected with either B3.13 or D1.1 genotypes [123,124]. A high similarity between strains isolated from a human case reported in Washington (A/Washington/240/2024) and D1.2 genotype strains isolated from pigs in Oregon was also noted, suggesting high potential for a zoonotic link between the swine and human cases [123].

Given the growing opportunity for zoonotic spillover and the severe clinical outcomes of human infections with the D1.1 genotype, enhanced surveillance in both farmed and companion animals, like domestic cats, is essential, as they may act as bridging hosts for cross-species transmission and accelerate the H5N1 evolutionary trajectory.

## 5. Conclusions

In the last two decades, only three clades of the GsGD H5 lineage spread globally—clades 2.2, 2.3.2.1, and 2.3.4.4b. Among these, clade 2.3.4.4b has demonstrated a unique capacity for extensive reassortment and host adaptation. It has spread rapidly across continents, reaching novel environments such as the Antarctic region, and infected a broad range of aquatic and terrestrial mammalian hosts. The advent of cow-to-cow and then cow-to-human transmission is alarming and signals potential for a new trajectory of avian influenza evolution. Continued global surveillance and genomic analysis are essential to monitor viral evolution and continuously reassess pandemic risk. Effective therapeutic and vaccination strategies must be developed in parallel for rapid response should the virus become capable of efficient human-to-human transmission.

## Data Availability

No new data were created in this study. Data sharing is not applicable to this article.
